# Medical Opinion and Movement

**Published:** 1912-01-20

**Authors:** 


### MEDICAL OPINION AND MOVEMENT.

### Beri-beri.

IN the *Philippine Journal of Science* for June 1911 Chamberlain and Vedder contribute a very interesting communication on the ætiology of beri-beri. Dealing with the polyneuritis produced in fowls by feeding on polished rice, the two authors have repeated the work done by Fraser and Stanton in the Malay States and have been able to confirm it. After a series of experiments, based on rather small numbers perhaps, they found that the polyneuritis of fowls is not prevented by adding to a diet of polished rice any of the following substances: potassium phosphate, potassium citrate, potassium carbonate, potassium chloride, magnesium phosphate, phytin, phosphoric acid, or phosphoric acid combined with potassium chloride. The neuritis-preventing substance in rice polishings is soluble, however, in cold water and cold alcohol, and the neuritis may be prevented by means of an extract of rice polishings containing only those substances soluble in cold water and cold alcohol. This extract contains—as far as is known at present—ash, phosphorus, pentoxide, nitrogen, and sucrose. Schaumann's idea that the multiple neuritis of fowls is caused by a lack of phosphorus compounds in the grain is probably incorrect. The neuritis-preventing substance contained in the rice polishings is capable of dialysis through a parchment membrane, so this means it is not colloid in composition. Its exact nature is of great interest, and Chamberlain and Vedder are going on with further experiments which may ultimately determine what it is.

### Puerperal Infection from Loeffler's Bacillus.

GIDE, in a Thèse de Lyon, calls attention to the fact that puerperal infection may at times be the result of the presence of Loeffler's bacillus. Usually the organism is not met with in pure culture, but associated with the streptococcus pyogenes. False fibrinous membranes are met with in the region of the vulva and are quite analogous to those seen in the throat in diphtheria of the mouth. Clinical examination usually allows of this type of infection being readily differentiated from those due to the streptococcus, but in any case bacteriological examination settles the question beyond doubt, especially in mixed infections of a severe type. Contagion is usually brought about by the physician treating an obstetrical case after a diphtheritic one. Treatment consists in that of diphtheria, viz. injection of anti-diphtheritic serum as soon as the diagnosis is clear, added to the usual treatment in cases of puerperal infection by the streptococcus. The patient must be isolated and great care taken in the disinfection of the hands, linen, and instruments.

### Disinfection of the Abdomen with Iodine.

PROPPING, while willing to acknowledge the sterilising properties of tincture of iodine, utters a word of warning in the *Zentralblatt für Chirurgie* about its use in abdominal surgery, basing his statements on the results of his own clinical experience, in cases of appendicitis. Out of seventy appendicectomies which he has performed after a preliminary skin disinfection with the drug, in six cases complications have ensued of intestinal occlusion as the result of kinking or adhesions, whereas out of three hundred similar operations performed before this method of disinfection was introduced, a similar complication arose in five cases only. The author does not believe that these results are matters of pure chance. Animal experimentation has shown the facility with which the tissues take up iodine. Twenty minims of tincture of iodine in physiological salt solution injected into the peritoneal cavity of a dog are sufficient to produce fibrinous adhesions between all the organs. He states, therefore, that tincture of iodine is dangerous for the peritoneum and for the intestines. If this drug be used to disinfect the abdominal skin, sterile dressings should always be interposed between the skin and the bowel drawn out from the wound, even when towels have been placed round the edge of the wound. Such towels are always impregnated to some extent with iodine from the skin. The bowel should be wrapped up in a sufficient thickness of dressings which have been wrung out in a warm solution of sodium chloride.

### Adenoid in the Infants.

PHARYNGEAL vegetations are commonly met with in infants. Durif, in a Thèse de Lyon, admits the relationship between rickets and these vegetations, both being the result of some chronic intoxication whether congenital or acquired. The clinical picture of the affection in its ordinary form is well marked, with a predominance of respiratory phenomena and alimentary disturbance. There exists, however, a latent variety in which certain symptoms, such as laryngeal spasm, asthma, and night-cough have to be recognised and traced to their actual cause. Prognosis is always overshadowed by the possibilities of grave troubles in the course of the evolution of the malady, as well as by the production of other redoubtable complications, especially those affecting the ears. Medical treatment is permissible if the adenoids are slight and no complications are present. Should the contrary be the case, treatment is of necessity surgical, though medical treatment may be tried first if the symptoms are not urgent. It may be necessary to remove the growths piecemeal at more than one sitting, according as to whether there is hæmorrhage or not, as well as according to the age of the little patient.

### The Sanitation of the Isthmian Canal.

COLONEL GORGAS in his monthly report of the Department of Sanitation of the Isthmian Canal Commission gives some very interesting details concerning the health of the workers employed there. For July 1911 the death rate per thousand was only 14.06, a very small figure when one considers the tropical nature of the country. Plague, smallpox, and yellow fever have been completely stamped out of the canal zone, and after this accomplishment the death rate becomes much as in America or England or other temperate climates. Malaria still exists of course and this levies its toll on the inhabitants, eight deaths from this cause alone having taken place in the month of July 1911. Amongst other diseases are noted nephritis, lobar pneumonia, blackwater fever, tuberculosis, typhoid, and diphtheria. Of the latter there has recently been a small epidemic, but generally speaking the type of the disease has been very mild and very few deaths have occurred. Such a satisfactory state of affairs must be credited to the great energy and sanitary ability of Colonel Gorgas. Without such a leader the work of making the canal could never have been undertaken.

### Fat Embolism of the Brain.

It has long been stated in text-books that after fracture of the long bones involving the marrow a transient condition of lipæmia results, with a tendency to the impaction of fat particles in capillaries first of the lungs and later in the systemic circulation; but it has fallen to the lot of few apparently actually to see such fat embolism demonstrated under the microscope, so that the importance of the condition is often forgotten. It requires special staining methods to demonstrate fat in the tissues, for the ordinary processes which are used in the preparation of microscopical sections dissolve out the fat globules, leaving them to be recognised, if they can be recognised at all, as empty spaces, not where fat is but where fat has been. By this means it has been shown that after accidents, especially those which include the fracture of bones, capillary embolism with fat globules occurs throughout the body, including especially the spinal cord and brain, and the importance of this in connection with the cerebral and spinal after-effects of railway accidents, to which the terms "neurasthenia" and "railway spine" have been applied, generally with a sort of feeling that the condition is more or less a neurosis, is obviously very great. It is only a question of degree for this fat embolism to be sufficiently extensive not merely to interfere with the functions of the nerve system, but to stop them altogether; in other words, one of the causes of death from fractures and other accidents is multiple capillary embolism, and this is a point which should be borne in mind when inquests are being made upon accident cases, for it may often be wise to have sections of the brain and other nerve structures prepared and specially stained by the Sudan III. process, without which the occurrence of even fatal fat embolism may pass unrecognised.

### Gout, Diabetes, and Tuberculosis.

It is an old observation that the gouty are but seldom the subjects of tuberculosis, but that diabetics are especially vulnerable to this infection. Dr. Nathan Raw, writing in the *Clinical Journal*, propounds the old diathetic view of these two mysterious disorders of metabolism, but he has experimental as well as clinical observations on which to found his belief. He took 10 c.c. of venous blood from a diabetic patient, and mixed it with glycerine agar. This was incubated at 37° C. for a few days to make sure of sterility, and then a pure culture of tubercle bacilli of the human type was added; at the same time a control tube containing glycerine agar only was inoculated. The two tubes were incubated for fourteen days: that containing the diabetic blood showed a most luxuriant growth, whereas the control tube contained only the usual moderate growth. The experiment was then repeated, using gouty blood instead of diabetic. This time no growth was seen at all at the end of four weeks, and even after eight weeks there was very little; the control tube showed the ordinary growth. The experiments were then repeated with blood from another case of diabetes and from another case of acute gout; the result was exactly the same as before. Dr. Raw deduces hence: (1) that diabetes predisposes to tuberculosis; (2) that gout has an inhibitory effect on the growth of tubercle bacilli; (3) that the blood-plasma is the determining factor in the growth or inhibition of bacterial infections within the human body.

### The Disintoxication Cure.

Dr. Bignon in *Le Nord Médical* has some remarks to make upon the therapeutic value of the treatment introduced by Dr. Guelpa, under the name of "La cure de désintoxication." It consists in a water diet for three or four days, with the object of getting rid of toxins and worn-out tissues of the body, and in repeated purgation to disinfect the intestine. The inventor recommends that a bottle of some purgative water be taken daily, lukewarm, for three or four days, or an ounce to an ounce and a half of castor-oil followed by a litre of "tisane" daily for the same period. During this time the patient is deprived of all food and only allowed boiled water or some feebly mineralised water or weak tisane, with or without sugar, the object being to increase the secretion of urine and the sudoriferous glands. Fasting is uncomfortable at first, producing lassitude and general fatigue, which are however relieved by the purgation, a fact which makes the inventor believe that hunger is not brought about only by the desire of the organism for material to repair its losses, but also by toxic substances accumulated in the intestines. The cure lasts four days, of which the first is the hardest to endure, and on which headache and vertigo may be experienced. After the cure the patient should take to a milk diet, then to one of milk and vegetables, before resuming his ordinary life. It may be necessary to repeat the treatment two or three times before lasting benefit is experienced. It has been found particularly beneficial in cases of glycosuria and diabetes, in which it has to be repeated three or four times at weekly intervals. It has also been tried with hopeful results in cases of epilepsy and certain ocular affections.

